# Age-Related Diseases and Driving Safety

**DOI:** 10.3390/geriatrics5040080

**Published:** 2020-10-19

**Authors:** Michael Falkenstein, Melanie Karthaus, Ute Brüne-Cohrs

**Affiliations:** 1 Institute for Work Learning and Aging (ALA), Hiltroper Landwehr 136, 44805 Bochum, Germany; 2 Leibniz Institute for Working Environment and Human Factors (IfADo), 44139 Dortmund, Germany; karthaus@ifado.de; 3 LWL University Hospital, Clinic for Psychiatry, Psychotherapy and Preventive Medicine, 44791 Bochum, Germany; ute.bruene-cohrs@rub.de

**Keywords:** aging, mobility, older drivers, sensory functions, motor functions, cognitive functions, age-related diseases, medication, cognitive assessment, driving assessment

## Abstract

Due to demographic changes, the number of older drivers is steadily increasing. Mobility is highly relevant for leading an independent life in the elderly. It largely depends on car driving, which is a complex task requiring a multitude of cognitive and motor skills vulnerable to age- related functional deterioration. The almost inevitable effects of senescence may be potentiated by age-related diseases, such as stroke or diabetes mellitus. Respective pharmacological treatment may cause side effects, additionally affecting driving safety. The present article reviews the impact of age-related diseases and drug treatment of these conditions on driving fitness in elderly drivers. In essence, we focus on diseases of the visual and auditory systems, diseases of the central nervous system (i.e., stroke, depression, dementia and mild cognitive disorder, and Parkinson’s disease), sleep disorders, as well as cardiovascular diseases, diabetes mellitus, musculoskeletal disorders, and frailty. We will outline the role of functional tests and the assessment of driving behavior (by a driving simulator or in real traffic), as well as the clinical interview including questions about frequency of (near) accidents, etc. in the evaluation of driving fitness of the elderly. We also address the impact of polypharmacy on driving fitness and end up with recommendations for physicians caring for older patients.

## 1. Introduction

As the number of adults aged 80 and older will increase by 61% between 2015 and 2030 [1], a rising number of senior and geriatric people will be driving vehicles. This may pose considerable traffic safety concerns. Senescence leads to a decline of sensory, motor, and cognitive functions even in healthy people, although these age-related changes differ individually [2,3]. Considering the driver information processing (DIP) model (an interplay of perception, decision, and reaction), fitness to drive is contingent upon our eyes, brain, and musculosceletal system [4]. Hence, age-related mental and physical changes can lead to problems for older drivers in dealing with challenging traffic situations [3]. On the other hand, driving is of particularly high importance for older drivers to maintain mobility and social participation. Older drivers rate independence in mobility, namely driving their own car, as very important, and the prospect of driving cessation as highly negative [5]. Driving cessation is associated with poorer physical, social, and mental health [6]. Thus, the benefits of driving to the aged individual must be balanced with the potential risk to self and others posed by an impaired driving fitness. However, many drivers can compensate for functional losses to a certain extent, thereby substantially improving driving safety [3].

Age-related deficits can be caused or exacerbated by age-related diseases and pharmacological treatment. During the ongoing process of aging there is an increasing likelihood of suffering from more than one—up to numerous—age-related disorders. In a German study on traffic- and health-related data relevant for transportation safety of older traffic participants (AGE-V3), two thousand older (50 years and older) and one thousand younger drivers (between 16 and 49 years) were asked about their diseases and their mobility behavior [7]. The older drivers reported diseases (and related medication) relevant to traffic safety much more frequently than younger drivers. Moreover, the older drivers were often unaware of the effects of the diseases and the medication on their ability to drive. In fact, the accident risk increases with the number of diseases [8]. Certain medication classes are associated with potentially adverse driving patterns and hence increased risks for motor vehicle accidents in older drivers [9].

Most of the common age-related diseases are likely to influence driving behavior, namely diseases of the sensory systems, diseases of the central nervous system, sleep disorders, cardiovascular diseases, diabetes mellitus, musculoskeletal disorders, and frailty.

Moreover, almost all aged individuals are taking drugs to treat somatic and/or psychiatric conditions, not only prescribed by physicians but often supplemented by over-the- counter-medications, generally not communicated to their physician. Numerous intrinsic and extrinsic factors, and in particular aging, can alter medication effectiveness and/or side effects among individuals. For example, renal clearance is reduced in higher age, which leads to a slower excretion of some medication. Furthermore, as older drivers may also drink alcohol, interactions between medication and alcohol is a concern. Another factor to consider is drugs, particularly cannabis, which is used by a growing number of older persons as an analgetic or for recreational purposes. Several studies have shown an impairment of driving-relevant functions due to cannabis, e.g., [10]. A French study on more than 4000 drivers found a higher risk of being responsible for a fatal motor vehicle accident (MVC) under the influence of cannabis [11]. On the other hand, a recent study found no difference in self-reported driving ability and driving errors between older cannabis users and non-users [12]. More studies with young and old drivers are needed to clarify this issue.

A survey of drivers 55 years and older found that 69% used one or more prescribed medications potentially impairing driving fitness, while 10% used at least five [13]. In a study on patients arriving to the hospital because of a car accident, more than 80% were taking analgesics and cardiovascular substances, 74% gastrointestinal and almost 50% psychoactive drugs [14]. Almost 30% of these medications were associated with an increased risk of accidents.

In the following, we will outline the impact of age-related disorders and frequently used pharmacological substances to treat these conditions on driving-related functions and driving behavior. The focus of our review is more on age-related diseases and less on treatment. Readers particularly interested in medication effects on driving may refer to [8,9,15]. Due to the broad scope, this review is meant to summarize state-of-the-art scientific results in a more selective rather than comprehensive approach. To find international literature, we used PubMed and Google Scholar, starting with 1987 and ending in 2020. Search terms included e.g., aging, old-age or aged drivers, elderly, automobile driving, driving safety, driving fitness, medication, motor vehicle crash, etc. In the first place, we included reviews relevant to each topic. Further, publications with findings from observational or interventional studies were considered, whereas case series or case reports or studies conducted using fewer subjects than 20 were excluded. If several papers were found published on a specific topic, we selected the more recent papers.

## 2. Diseases of the Sensory System

### 2.1. Chronic Diseases of the Visual System

With increasing age, various visual functions such as visual acuity, twilight vision, contrast vision, color vision, as well as peripheral and stereo vision gradually deteriorate [3,16]. Certain eye diseases occur frequently in old age, namely: cataracts, glaucoma, and age-related macular degeneration (AMD) [17].

In the AGE-V3 study mentioned above, the older respondents named cataracts as the most common eye disease [7]. About half of Americans aged 65 to 74 years may have cataracts [18]. Usually, cataract symptoms become noticeable when driving a car. Cataracts often cause foggy vision. During darkness, light sources and oncoming headlights cause glare, and more so in older drivers [19]. The risk of accidents for drivers with cataracts is more than twice as high as in healthy drivers [20]. Night vision enhancement (NVE) systems may be able to mitigate problems with mesopic and night vision, as they provide the driver with information that is difficult for him to see in twilight or the dark, for example, pedestrians or traffic signs. However, previous findings on the usefulness of this technology for older drivers are contradicting [21], showing that the additional information may increase the workload of older drivers [22]. Hence further research is required on this topic. Nowadays, surgical methods are available to cure cataracts, which results in a significant reduction in vision-related driving problems [23,24].

Glaucoma is another common age-related disease of the eye. It is a leading cause of visual field loss in older populations [25]. The global prevalence of glaucoma for the population aged 40–80 years is 3.54% [26]. Overall, 20% of glaucoma patients experience progressive visual field loss despite receiving appropriate treatment, which may be unnoticed in the early stages of the disease but may nevertheless impair driving ability. In a population-based study, older drivers with glaucoma had an approximately 65% higher rate of at-fault MVC involvement in the prior five years than those without glaucoma [27]. Depending on the visual field defects’ location, the risk of accidents for drivers with glaucoma is increased [28]. In particular, visual field defects in lower or left areas appear to increase accident rates [29]. Even glaucoma patients with minor visual field failures may have considerable difficulties in driving tests [30]. A driving simulator study [31] found similar results: The number of accidents in the glaucoma group was correlated with three Goldmann visual field measures. The authors conclude that a visual field reduced to less than 100° of horizontal extent may place glaucoma patients with peripheral field loss at greater MVC risk. The glaucoma group also reported having had at least one car accident within the past five years. According to a study by Wood & Black [17], older drivers with glaucoma with even mild to moderate field loss exhibited impairments in driving ability, particularly during complex driving situations that involved tactical problems with lane positioning, planning ahead, and observation.

Glaucoma patients with additional cognitive and motor disorders have a strongly increased risk of accidents [28]. Deteriorations of subjective and objective driving skills were noticed in glaucoma patients who were assessed with different methods, namely driving behavior observations, behavior in the driving simulator, and vision tests [32]. Some glaucoma patients have learned strategies for compensation, such as more frequent eye movements or head turnings. However, recent research on the efficiency of such compensation mechanisms and their successful use in real traffic is still insufficient. Nevertheless, glaucoma often prompts the elderly to give up driving [33].

The third age-related disease of the visual system is age-related macular degeneration (AMD), which leads to a loss of central visual acuity. As expected, there are pronounced impairments of driving behavior among persons with AMD. In a recent study, Wood and colleagues assessed the on-road driving performance in 33 older drivers with early and intermediate AMD and 50 age-matched controls [34]. Drivers with AMD were rated as less safe than controls; safety ratings were associated with AMD severity. Drivers with AMD made more critical errors and exhibited more observation, lane-keeping, and gap selection errors and made more errors at intersections. The authors conclude that AMD can induce driving impairment, particularly during complex situations. Drivers with AMD avoid longer journeys as well as driving at night and in unfamiliar areas [35]. Interestingly, patients in the intermediate stage of AMD show better driving behavior than patients in the early or late stages [36]. This suggests a compensatory adjustment of driving behavior in the intermediate stage, which is not necessary in the early stage, and no longer possible in the late stage.

In addition to this triad of age-related eye diseases, diabetic retinopathy should be mentioned as a further traffic-relevant disease of the sensory organs, which is mentioned below in the section on diabetes.

Given the research results described, it is obvious that the usual testing of visual acuity is insufficient for assessing the visual problems of older and impaired drivers and should be supplemented by additional tests, which have to include a systematic measurement of the visual field (perimetry) but also measurements of contrast sensitivity, stereoscopic vision, color vision, and mesopic vision [16].

Some medications used in ophthalmology may have side effects that impair driving safety, such as pupil narrowing agents (miotics) that can cause tear flow, nausea, or disturbances when the eye is adjusted to a changed situation. Especially during driving at twilight or night, when older people have problems anyway, such medication limits driving ability. In the case of central lens opacity, as frequently seen in older people, miotics impair vision even more.

In summary, age-related eye diseases, in particular glaucoma, can lead to massive impairment of the fitness to drive a car, especially due to disease-related reductions in visual acuity, mesopic vision, and visual field restriction. Hence, exclusive testing of visual acuity is insufficient and should be supplemented by additional tests. Mildly reduced visual acuity may be remediated using corrective glasses. Drivers with mild visual field defects can be instructed to use panoramic mirrors, compensatory head and eye scanning, or other driving strategies [37]. In the case of a chronic progressive loss of visual acuity or loss of field of vision, the physician should give information and educate the patient at an early stage of the disease concerning an impending inability to drive along with a regular medical re-evaluation.

### 2.2. Diseases of the Auditory System

Age-related hearing impairment is widespread in older people, and prevalence estimates range from 20.6% in adults aged 48 to 59 years to 90% in adults older than 80 years [38]. Since some important information in traffic is acoustic, such as engine noises that signal a vehicle’s approach or sirens of an emergency vehicle, severe hearing loss should be associated with an increased risk of accidents. Aging also contributes to changes in spatial hearing, such as the ability to localize sound sources. Further, older subjects show poorer performance on binaural tasks that require precise temporal processing (e.g., [39]) such as lateralization and localization tasks, as well as in the detection of signals in noise [40,41]. All these aspects of hearing are relevant for safe navigation in everyday road traffic. Despite this obvious importance of hearing for driving, there are only very few studies on age-related hearing impairment/loss and driving safety. A good overview of the related literature has been provided by Edwards and colleagues [42]. They found mixed results in studies with self-reported hearing impairment. Some studies found no associations between self-reported hearing ability and driving safety, e.g., [43,44]. However, in a large study with 3654 drivers (in left-hand traffic), self-reported hearing impairment in the right ear was associated with increased accident rates [45]. This suggests that an impairment of the ear directed to the traffic impairs driving safety. In the two available studies mentioned by Edwards and colleagues that used objective hearing measures, adverse effects on driving were found. In the first study, older drivers with moderate to severe hearing impairment demonstrated poorer driving performance in the presence of distracters than those with normal or mild hearing impairment [46]. The second study on 4.603 × 10^4^ male workers employed in noisy industries showed that bilateral hearing loss enhanced the prevalence of accidents [47]. However, two studies are not enough to conclude an enhanced risk for older drivers with hearing impairment, and no further studies on this topic were found. Individuals with hearing loss often show compensatory behavior such as driving at lower speeds, using a more comprehensive visual search behavior and being less engaged in distracting activities, which likely mitigates driving problems due to poor hearing [48]. Older drivers with a combination of visual and hearing impairment (dual sensory impairment) reported more driving difficulties [49] and a greater restriction of driving skills [43] than patients with isolated visual or hearing disorders or no sensory impairments. Clearly, more research on the impact of hearing problems and dual sensory impairment on driving behavior and safety is needed.

Thus, a regular examination of the auditory system in the elderly is indispensable. This should include not only pure tone audiometry but also spatial hearing and the ability to detect signals in a noisy background or when distracting visual stimuli are present. Mild to moderate monaural or binaural hearing deficits may be remediated using hearing aids. In certain severe cases of bilateral sensorineural hearing impairment cochlear implants can be used. Such implants may also improve cognitive domains that are relevant for driving [50].

## 3. Diseases of the Central Nervous System

### 3.1. Stroke

Strokes are based on a sudden disturbance of central nervous circulation, caused by ischemia in 80% of the cases. The incidence of stroke rapidly increases with age, doubling for each decade after age 55 [51]. A stroke is associated with physical, sensory, and cognitive functional impairments that affect driving. Driving performance can be impaired by hemiparesis, sensory disorders in the arms and legs, and spasticity. Furthermore, visual field reductions and driving-relevant cognitive disorders of attention, orientation, and ability to make decisions may occur.

Stroke patients are almost twice as likely to cause an accident as healthy people, irrespective of their medical treatment [52]. Stroke patients can suffer from a variety of different symptoms and undergo different invasive and non-invasive treatments. This means that driving fitness cannot be determined in a generalized manner, but has to be assessed by specific psychiatric and neuropsychological tests, and by a practical driving test comprising complex situations. For example, a Canadian driving simulation study found that patients with ischemic stroke (IS) and patients with subarachnoid hemorrhage (SAH) had problems with lane maintenance [53]. Only IS patients showed additional difficulties with speed maintenance, whereas SAH patients showed additional problems in transposition maneuvers. In a German study, 116 patients with predominantly vascular and traumatic brain damage underwent an extensive driving test in real traffic [54]. In total, 42% of the patients failed the driving test, mainly due to visual field impairments.

In addition to strokes, transient ischemic attacks (TIA), defined as a brief episode of neurological dysfunction caused by ischemia in the brain, often are accompanied by impaired consciousness or neuropsychological symptoms impairing driving safety. Silent brain infarction (SBI), meaning the absence of clinically obvious symptoms only diagnosed by MRI, which is linked to a two-fold increased risk of future stroke [55] often leading to a cognitive decline and dementia, might have an impact on driving fitness [56].

Due to a German study, the most common post-stroke cognitive deficit limiting the ability to drive is an impairment of attention [57]. In that study, a neuropsychological assessment revealed that 52% of the patients with ischemic stroke and about 60% with hemorrhagic strokes were unfit to drive, which is in good agreement with [54]. In a study on cognitive deficits of stroke patients and their performance in an on-road driving test, 37% of the patients showed poor driving performance, and divided attention emerged as the main determinant of the on-road driving performance [58]. Deficits of executive functions following strokes are relevant for impaired driving fitness [59]. In a Swedish study, 35 of 78 patients with stroke or traumatic brain injury failed an on-road driving test [60]. Furthermore, the authors showed that driving test results could be predicted in more than 80% of the patients by a combination of three neuropsychological tests. Nevertheless, driving behavior observations are indispensable, since stroke patients can show comparable performance to healthy controls in driving simulation and on-road tests due to experience and compensation strategies [61]. Thus, the driving fitness of stroke patients should be determined with a combination of neuropsychological tests and driving behavior observations in real traffic.

Stroke can also occur acutely while driving, which is the case in 4% of all strokes, leading to accidents in 16% of these instances [62].

Stroke patients are at risk for future ischemic attacks, which are difficult to predict in the individual patient depending on risk factors and medical treatment. According to prospective studies, recurrent stroke rates range from 6–13% within the first year after the first stroke event and 5–8% per year in the following 2–5 years [63]. Clinical and neuropsychological test results have to be considered when stroke patients who want to resume driving seek advice about their crash risk [64].

Counseling of post-stroke patients has to keep in mind that cognitive deficits may even worsen in the years following an incident stroke. Levine and colleagues [65] followed up on cognitive functions of 2.3572 × 10^4^ stroke patients after six years and found that even without intermediate re-stroke, cognitive decline was accelerated compared to controls, especially for executive functions. These findings support data published by Sachdev and colleagues [66]. They assessed 123 patients with stroke or TIA to determine the profile of cognitive decline over one year. Compared to age-matched healthy controls, the patients had a greater decline in verbal memory and certain visual functions even in the absence of an interval stroke.

In summary, strokes can lead to traffic-related impairments in motor, sensory, and cognitive functions. Above all, impairments of attention and field of vision can affect the ability to drive. Stroke patients are more likely to fail an on-road driving test and cause an accident than healthy people. Stroke patients should be tested regularly with neuropsychological test batteries comprising driving-relevant cognitive and visual functions.

### 3.2. Depression

Depression is a common disease in old age. Major depression occurs in 2% of adults aged 55 years or older, and its prevalence rises with increasing age. Overall, 10% to 15% of older adults have clinically significant depressive symptoms, even in the absence of major depression [67]. In a sample of people over 60 years in Sweden, a prevalence of about 6% was found, with the largest proportion of patients suffering from mild stages of depression [68]. In adults 75 years and older, the prevalence of depressive disorders ranges from 4.5% to 37.4%, depending on study design, sampling strategy, and applied diagnostics [69]. Further, many older patients with depression suffer from other medical conditions impacting driving safety [70]. According to a Dutch study, almost half of the depressed elderly still showed symptoms of depression at a 2-year follow up, indicating an often-chronic course of the disease [71].

Major depression (MDD) impairs cognitive functions, e.g., memory and executive functions [72]. Among those, executive functions are most important for safe driving. In a meta-analysis on 113 studies that compared participants with MDD to healthy control participants, MDD was reliably associated with impaired performance on neuropsychological measures of executive functions, with effect sizes ranging from 0.32 to 0.97 [73]. Late-life depression may be related to a deficit of inhibitory control, a core executive function [74]. Importantly, deficits in executive functions appear to increase with each depressive episode and persist after symptom remission [75]. The signature of cognitive impairment in depressed older patients with early-onset bipolar disorder also depicts mainly executive dysfunction [76].

Wickens and colleagues [77] reviewed the existing literature on the association between depression and driving performance. The majority of the epidemiological studies identified depression as a contributor to increased collision risk. Some driving simulator studies showed that depression impairs driving behavior. For example, non-medicated MDD patients exhibited slower steering reaction times and a greater number of crashes than control participants [78]. A later review of 19 epidemiological studies found that both depressions per se and antidepressant medication reduced driving safety [79]. The estimates of the odds ratio (OR) of crash involvement associated with depression ranged from 1.78 to 3.99. Furthermore, the use of antidepressants was associated with an approximately 40% increased MVC risk.

Although tricyclic antidepressants (TCA) such as amitriptyline, imipramine, or doxepin have been widely replaced by better-tolerated agents, they are still in use. They can produce orthostatic hypotension and varying degrees of sedation. Leroy and Morse [15] found a 41% greater likelihood of motor-vehicle crashes when using TCA. A dose-dependent association was found by patients using more than 125 mg amitriptyline per day [80]. Iwamoto and colleagues [81] showed that even four hours after administering 25 mg of amitriptyline, drivers showed more lateral weaving and variation in car-following distance. For selective serotonin reuptake inhibitors (SSRI), the impact on driving safety is much lower. In a meta-analysis, Ravera and colleagues [82] concluded that SSRIs only appear to impair driving when given in high dosages. Well-chosen modern antidepressants can even improve driving performance in patients with depression. For example, Brunnauer and colleagues investigated the influence of two antidepressants (reboxetine or mirtazapine) on psychomotor functions, stress tolerance, and driving performance in a simulator in 40 in-patients with depression [83]. The data were collected before and 7 and 14 days after the start of the medication. Before taking the medication, 65% of the patients failed to meet the minimum requirements for driving. Fourteen days later, the measured functions were significantly improved in both treatment groups compared to a healthy control group, and 80% of the patients now passed the driving test. In a later study by those authors [84], 40 depressed patients were treated with agomelatine or venlafaxine. Psychomotor tests were performed before, as well as 14 and 28 days after the start of treatment; besides, the patients completed a driving test on the 28th day. Again, both medicated patient groups showed an improvement in psychomotor skills and stress tolerance compared to a healthy control group, and approximately 73% of the patients were classified as fit to drive. However, the control group still outperformed the patients in the functional tests.

Studies on moderate or minor depression and cognition or driving fitness are scarce. The few available studies suggest that also moderate or minor depression is related to an impairment of cognitive functions relevant for driving. For example, individuals with dysthymia showed pronounced difficulties in mental flexibility, which is certainly relevant for driving [72]. Mild and subclinical depression appear to influence the processing of feedback [85] and impair behavioral adjustments after negative feedback [86] which are functions clearly relevant to driving. The mere induction of sadness or moderate/minor depression was found to narrow spatial attention [87], which may impair driving safety because peripheral objects could be overlooked. In the elderly, alcohol consumption is a frequent comorbid problem to depression [88], although research on its impact on driving safety in the elderly is still lacking.

While it is clear that depression may impair driving, there is also evidence that the restriction or loss of mobility in older people is conducive to depression [89], which has to be considered when thinking about driving cessation. For example, 2–3 years after giving up driving, older people showed more depressive symptoms than active drivers [90]. A recent meta-analysis revealed that driving cessation in older adults nearly doubles the risk of depressive symptoms [91].

In summary, depression impairs cognitive functions relevant for driving, which consequently leads to an impairment of driving safety. However, well-chosen antidepressants can significantly improve driving performance. On the other hand, a loss of mobility can lead to depression, which underlines the importance of driving for older people.

### 3.3. Dementia and Mild Neurocognitive Disorder (NCD)

Dementia, or severe neurocognitive disorder (severe NCD), is one of the most consequential diseases of old age. Around 50 million people worldwide are suffering from dementia, and around 10 million cases are added each year [92]. Depending on the region, the prevalence rates vary from 4.6% (Europe) to 8.7% (North Africa) [93]. The most common dementia disorder is Alzheimer’s disease (AD), a neurodegenerative dementia, which accounts for about two-thirds of new diagnoses. The second most common type is vascular or mixed type dementia, followed by dementia with Lewy bodies (DLB), frontotemporal dementia (FTLD), and others [94]. Similar to Parkinson’s disease, dementia can lead to multiple functional, mainly cognitive, impairments. In addition to memory disorders, attention disorders and executive deficits often occur [95]. Neurodegenerative dementias usually have a progressive course, so impairments continuously increase.

Several cognitive functions disturbed in dementia are essential for driving, in particular executive functions and attention [95]. Already Parasuraman & Nestor [96] pointed out that traffic accidents are associated with attention, especially shifting of attention, and that these functions are impaired even in the early stages of AD, which results in an increased risk for drivers with AD. Later studies confirmed relationships between attention deficits and impaired driving skills in Alzheimer’s patients [97].

Nevertheless, many patients with dementia continue to drive, although showing impairments of driving behavior [98,99,100,101]. Fitten and colleagues compared driving behavior in real traffic in five groups of test subjects: patients with mild AD, patients with vascular dementia, patients with diabetes, healthy older adults of the same age, and healthy young adults [98]. Both dementia groups showed poorer performance than the diabetes group and the two healthy control groups. The performance did not differ between the two dementia groups or the three control groups. Uc and colleagues [99] reported that Alzheimer’s patients made more turning mistakes, lost orientation, got lost, and were more likely to jeopardize road safety with their maneuvers than cognitively healthy drivers. Hoggarth and colleagues [101] administered a sensory-motor and cognitive test battery and an on-road driving test to almost 300 patients with different types and levels of cognitive impairment. More than half (56%) of the patients failed the driving test. The test battery results correctly classified 75.6% of the sample into on-road pass and fail groups. A meta-analysis of 32 studies with Alzheimer’s patients and healthy controls [102] showed that about 14% of patients with very mild dementia and 33% with mild dementia failed an on-road driving test, while only 1.6% failed in the control group. Cognitive tests revealed that executive functions, attention, spatial thinking, and general cognition were significant predictors of driving performance, with the Trail Making Test (Parts A and B) being the most reliable.

Rizzo and colleagues [97] examined 39 older drivers, including 21 with Alzheimer’s dementia in the driving simulator. Six of the drivers with dementia were involved in accidents during the simulated drive, but none of the healthy controls. In particular, Alzheimer’s patients experienced near-accidents almost twice as often as healthy people. Predictors of the increased probability of accidents were the impairment of spatial-visual attention and spatial thinking. On the other hand, most of the patients were not involved in accidents, indicating that they may have developed compensation processes.

While vascular, and especially Alzheimer’s, dementia is well studied concerning driving ability, the importance of frontotemporal dementia (frontotemporal lobar degeneration, FTLD) [100,103] is underestimated. FTLD is the second most common cause of neurodegenerative dementia in the elderly; their prevalence is estimated at 3–15 per 1 × 10^4^ people over 50 [100]. In a survey of relatives of patients with Alzheimer’s dementia and FTLD, changes in driving behavior were observed in 90% of the patients with FTLD compared to 58% of the patients with Alzheimer’s dementia [95,100]. While Alzheimer’s patients primarily had orientation problems, the patients with FTLD showed an aggressive, risk-taking driving style with conspicuously frequent traffic rule violations. In total, 37% of patients with FTLD had been involved in an accident since the onset of the disease, compared with 19% of Alzheimer’s patients. While most of the patients with Alzheimer’s dementia were clear about their changes in driving behavior, most patients with FTLD were not and refused to give up driving. This lack of patients’ awareness of deficits poses a high risk, which is why the authors recommend that patients with FTLD should stop driving as soon as possible.

Obviously, the severity of dementia is crucial for fitness to drive. A person with moderate to severe dementia is no longer fit to drive, whereas driving ability may be maintained in mild AD for some time [104]. A meta-analysis by Hird and colleagues found on-road fail rates of 33% for mild AD, and about 13% for very mild ADD, compared to 1.6% in healthy controls [102]. However, the crash rate of patients with mild and very mild dementia might be the same as for healthy controls, which suggests compensatory mechanisms [105].

A preliminary stage of Alzheimer’s disease is mild cognitive disorder (MCI), and in particular amnestic mild cognitive impairment, aMCI, with a high predictive value of a conversion to AD of about 30% in 3 years [106]. In MCI, cognitive deficits can occur in a wide variety of areas, such as memory or executive functions. Patients with MCI score lower on off-road and on-road assessments compared to healthy controls but are less impaired than patients with Alzheimer’s disease [107]. Hird and colleagues [108] compared the performance of MCI patients and healthy controls in a driving simulator. MCI patients committed twice as many driving errors and had greater problems with lane-keeping and turning left than healthy subjects. MCI patients with multiple-domain mild cognitive disorders (md-MCI) showed the greatest impairments. The authors suggest differentiating between subgroups when assessing the fitness to drive of MCI patients.

Piersma and colleagues investigated which of three off-road measures, namely a clinical interview, a neuropsychological assessment, and a driving simulator ride, would best predict the fitness to drive of patients with AD [109]. The criterion fitness to drive was determined by an on-road driving assessment. The authors concluded that all three types of off-road measures were equally predictive of on-road driving performance, and combining all three types yielded a predictive value of 92.7%. In a further study, they compared patients with different types of neurodegenerative diseases (AD and non-AD dementias as well as PD) with healthy controls [110]. They used off-road measures such as clinical interviews and neuropsychological assessments; again, driving fitness was assessed in real traffic. Patients with AD performed significantly worse than healthy older drivers on operational, tactical, visual, and global aspects of on-road driving. In patients with AD, on-road measures were significantly associated with off-road measures. These results show that a combination of off-road measures may render costly real driving unnecessary for the prediction of driving fitness in AD.

Recently, Toepper & Falkenstein [95] provided a comprehensive overview of the impact of different forms of dementia on driving behavior. The authors stated that driving fitness is severely impaired in moderate and severe dementia, irrespective of the type of dementia. In milder disease stages, fitness to drive appears to be more impaired in non-Alzheimer dementias than in AD, since the non-Alzheimer syndromes are not only associated with cognitive, but also non-cognitive risk factors, such as behavioral or motor symptoms. Patients with MCI may still be able to drive depending on the presence of compensation mechanisms.

Hence, to conclude on driving restrictions in dementia and MCI, a proper diagnosis of the underlying neurocognitive disorder is indispensable, as differing underlying pathologies have a differential impact on driving safety and hence on the physician’s advice to their patients. Therefore, a multifaceted neuropsychological assessment should be performed, including relevant aspects of attention, executive functions, and visuospatial skills. Physicians should counsel patients with moderate and severe dementia and frontotemporal dementia by explaining the high risk for driving. Regular, at least yearly, and in rapidly progressive cases even more frequently, follow-up examinations should take place. Specialized institutions may support general practitioners in the diagnostic work-up and counseling of patients and their relatives. As to our knowledge, no research has been done on anti-dementia drugs and its effects on driving fitness so far.

### 3.4. Parkinson’s Disease

Parkinson’s disease (PD) is one of the most frequent neurological diseases of old age. The number of individuals over age 50 with PD in the most populous countries was between 4.1 and 4.6 million in 2005 and will double to between 8.7 and 9.3 million by 2030 [111]. In PD, dopamine-producing neurons in the substantia nigra degenerate, causing a lack of dopamine. This results in various physical, cognitive, and psychological impairments, which can harm the ability to drive. PD is defined primarily as a movement disorder, with the typical symptoms being resting tremor, rigidity, bradykinesia, and postural instability. The most common non-motor symptoms are impairments of executive, attentional, and visuospatial functions. Each of these restrictions alone leads to a loss of ability to drive and, even more so, their combination. In particular, the cognitive deficits in PD may affect driving, such as deficits of attention, visual memory, spatial thinking, inhibition, and task switching [112]. Executive impairments show up, for example, when automatic action tendencies have to be suppressed, which makes it more difficult to deal with complex and surprising driving situations and may result in impaired action adaptation and learning from driving errors. Furthermore, many Parkinson’s patients show sleep disorders and increased daytime sleepiness, the latter causing high risks for drivers [113,114].

More than 80% of Parkinson’s patients have a driver’s license and most of them are active drivers. Due to the multiple deficits in Parkinson’s disease, a limited driving ability can be expected. In a survey of 361 Parkinson’s patients, 15% of them had been involved in an accident in the past five years, 11% of whom were at fault [115]. The accident rate was higher among those who reported drowsiness and sudden sleep attacks while driving. Wood and colleagues assessed the driving performance of 25 patients with idiopathic PD and 21 age-matched controls in real traffic [116]. The drivers with PD were rated as less safe than controls, and more than half of the drivers with PD would not have passed an official driving test. The driver safety ratings were more strongly related to disease duration than disease severity. Drivers with PD made more errors than healthy subjects during maneuvers that involved changing lanes and lane-keeping, monitoring their blind spot, reversing, car parking, and traffic light-controlled intersections. In a study by Uc and colleagues [117], the most frequently observed error categories in PD patients were lane observance, turns, lane change, stop signs, speed control, and turn errors. An off-road battery of cognitive, visual, and motor tests predicted safety error counts within the PD group. The main reasons for driving errors in this study were impairments of different visual and cognitive functions. However, there were strong inter-individual differences in driving performance and some patients showed normal driving behavior. Familiarity with the driving environment was a mitigating factor in drivers with PD.

In a meta-analysis on the driving-related behavior of PD patients, driving problems were found for patients at all levels of driving ability [118]. Some of the patients showed strategic compensation mechanisms that improved driving performance. The authors therefore recommend practicing such strategies. Additionally, Parkinson’s patients could benefit from the support of a lane departure warning system that Eby and colleagues [21] consider as useful for older drivers. However, up to now, there are not enough studies on this topic to make a reliable recommendation.

Not all studies showed relevant driving impairments in PD: Singh and colleagues [119] assessed the driving ability of 154 individuals with PD referred to a driving assessment center by a combination of clinical tests, reaction time, and in-car driving test. The majority of patients (66%) were able to continue driving, although 46 individuals required an automatic transmission and 10 others needed car modifications. Dosage of medication and length of driving history were not related to the driving test scores.

Medical treatment of PD aims at compensating dopamine deficiency in the brain to improve motor skills and cognitive functions and therefore improve driving ability [120]. The most effective drugs currently available are levodopa and dopamine agonists. Various side effects of medication used for PD are relevant for driving safety, e.g., unwanted motor effects, particularly frequent in patients receiving levodopa. Disease progression and long-term treatment with levodopa lead to “wearing-off” symptoms, dyskinesias, and other motor complications in up to 80% of patients. Fluctuations in mobility, dyskinesia, dystonia, and excessive and abnormal involuntary movement may occur during treatment with antiparkinsonian medication. Complications range from the predictable “end-of-dose” deterioration or the “wearing-off” phenomenon to the “on-off” phenomena with very sudden swings from mobility to immobility [121].

Further, Parkinson´s disease patients tend to suffer from sudden sleep attacks, posing a substantial hazard to driving. Such sleep attacks either have a sudden onset without warning or a slow onset with prodrome drowsiness. Evidence suggests that these sleep attacks more often occur to PD patients on dopamine agonists (either levodopa, ergot, or non-ergot agonist). Scopolamine, synthetic anticholinergics, and bromocriptine may lead to microsleep and an impairment of impulse control [13]. A more invasive treatment option, deep brain stimulation (DBS), appears to have a positive effect on driving safety of PD patients [122]. In that study, patients with DBS performed better than patients without stimulation. Compared to healthy people, the patients treated with DBS drove more slowly and carefully, but with similar safety.

In summary, patients with Parkinson’s disease show a significantly reduced driving safety due to motor, sensory, and cognitive impairments. However, familiarity with the environment and strategic compensation mechanisms can mitigate driving impairments in PD. Physicians should bear in mind that PD is a progressive disease often associated with comorbidities, meaning driving fitness has to be reevaluated on a regular basis. Medication should be selected with regard to side effects and appropriateness for patients who want to continue driving.

## 4. Sleep Disorders

### 4.1. Chronic Insomnia

On German highways, about a quarter of all accidents occur because the driver falls asleep for a short time [123]. One of the reasons for fatigue is insufficient sleep. Sleep disturbances are a common symptom of elderly people. Behavioral factors and primary psychiatric disorders are also affecting sleep in this population. Otherwise healthy older people often show poor sleep patterns; the duration of sleep is shortened and the waking time is extended. Circadian rhythms are altered, and deep sleep phases in particular are becoming significantly shorter [124]. Poor duration and quality of sleep can lead to fatigue and cognitive decline [125,126], both affecting the ability to drive. Already in 2000, Masa and colleagues [127] reported that 33% of drivers are habitually sleepy while driving and report falling asleep frequently while driving. Such drivers have a 13-fold increased risk of having an automobile crash than control subjects. A high proportion of habitually sleepy drivers had an unrecognized respiratory disorder during sleep. The investigators concluded that a respiratory sleep disorder is an independent risk factor for crashes in habitually sleepy drivers. Different kinds of chronic insomnia are associated with motor vehicle crashes in the general population [128].

As to medication against poor sleep, benzodiazepines, which are also prescribed for sleep disorders, should be mentioned above all. Benzodiazepines are associated with a five-fold increase in the risk of accidents [52], and more so for men than for women [129]. Driving under the influence of benzodiazepines is assumed to be as impaired as 0.5‰ blood alcohol [130]. The effects and side effects of benzodiazepines were also associated with other driving problems, including slower reaction times and problems with steering, speed control, and observing the surrounding traffic [131]. In a review article, Verster and colleagues [132] summarized residual effects of sleep medication on driving behavior in real traffic. Benzodiazepines that are taken in the evening as a sleep aid impair driving behavior still the following morning and sometimes even 16–17 h after ingestion. Similar drug-induced impairments were found in cognitive performance tests, and when driving in the simulator. Therefore, benzodiazepines with a longer half-life, which are associated with significantly unsafe driving behavior [133] and an increased accident rate [134], are considered to be particularly risky. Since the half-life of drugs increases with age, this is of particular importance for older people [135]. Other medications that are prescribed for sleep disorders can impair the ability to drive, such as the so-called z-substances (Zolpidem, Zaleplon, Zopliclone) [13,136]. For example, Zolpidem has consistently been shown to be associated with an increased risk of motor vehicle collision [137].

### 4.2. Sleep-Related Breathing Disorders

An important cause of daytime sleepiness is sleep-related breathing disorders. One of the most important sleep disorders is obstructive sleep apnea syndrome (OSAS), in which there are frequent pauses in breathing during night sleep caused by a brief collapse of the upper airways. Due to the resulting short-term lack of oxygen, the deep sleep and the dream phases are shortened. While younger people rarely suffer from OSAS, the elderly are increasingly affected: From the age of 60, approx. 26% of women and more than 50% of men suffer from OSAS. The prevalence of at least moderate OSAS in older people varies from 7% and 44% in several studies using different methods, with men being affected significantly more frequently than women [138]. The disease can lead to an urge to sleep and nodding off, especially in monotonous situations and during periods of reduced alertness (between 2 a.m. and 5 a.m. and around 2 p.m.). OSAS leads to impairments of important cognitive functions such as attention, and hence an increased accident risk [139,140,141]. Patients with untreated OSAS show poorer driving performance in real traffic [142] and in the driving simulator [143] than patients without sleep apnea. Moreover, several studies found a relation between OSAS and traffic accidents (e.g., [144]), which appears to be more pronounced in non-professional than in professional drivers [145]. New European Union regulations state that untreated moderate to severe OSAS with excessive daytime sleepiness (EDS) constitutes a medical disorder leading to unfitness to drive [146]. However, fitness to drive can be well re-established by a treatment with a sleep mask that puts continuous pressure on the upper respiratory tract (CPAP; [140,147]. The ability to drive is usually restored within six weeks after the initiation of CPAP therapy [140]. Tests with driving simulators could reveal impairments of cognitive functions and driving behavior in OSAS patients, as well as their improvement through CPAP even after two weeks [140]. Similarly, Findley and colleagues [147] could show that patients who were treated with CPAP had a lower crash rate during treatment than before (0.07 versus 0 crashes per driver per year). Finally, a recent meta-analysis of 10 studies demonstrated a significant reduction of motor-vehicle crashes in OSAS patients using CPAP masks [148]. Smolensky and colleagues [149] provide a detailed overview of sleep disorders, chronic medical conditions, and their risk for drowsy-driving road crashes.

In summary, age-related sleep disorders have a strong impact on driving fitness. Chronic insomnia can lead to fatigue and cognitive decline which both affect the ability to drive and the rate of accidents. Sleep-related drugs have side effects that mostly affect driving safety. Sleep-related breathing disorders impair cognitive functions and have strong effects on driving fitness, but can be well treated with sleep masks (CPAP). Physicians should advise and inform patients to achieve adherence to this treatment strategy, a major issue in the management of OAS. As patients tend to overestimate their usage of CPAP, objective monitoring comes into place [146].

## 5. Cardiovascular Diseases

Cardiovascular diseases are very common in the elderly. By far the most frequently reported disease is high blood pressure (hypertension), followed by cardiac arrhythmia, cardiac insufficiency (heart failure), coronary artery disease, heart attack, and angina pectoris. All of these diseases have a wide variety of different symptoms that can adversely affect driving ability and road safety [150].

### 5.1. Arterial Hypertension

Systemic arterial hypertension (AH) is the most prevalent chronic disease. It is defined as a blood pressure of more than 140/90 mm Hg without taking medication; controlled hypertension is defined as a blood pressure of less than 140/90 mm Hg while taking antihypertensive drugs. It is estimated that more than 31% of people worldwide had hypertension in 2010, with more than 75% affected in the age group 70 and over [151]. Hypertension is only associated with driving risk if it is extremely high. With very high blood pressure, the risks of heart failure and cerebral hemorrhage increase. The current German driving guidelines [152] state that group 1 (private car) drivers with a constantly increased diastolic value above 130 mm Hg are not able to drive. Such high diastolic values are rarely observed. For group 2 (professional truck, taxi, and bus drivers) the limit for safe driving is set to a diastolic value of 100 mm Hg. Hence professional drivers have to check their blood pressure regularly. This is all the more important as male bus drivers appear to have higher rates of hypertension than comparable groups of non-professional drivers [153].

The vast majority of patients with hypertension, especially those with well-controlled hypertension, are fit to drive. However, medication with antihypertensive drugs is not entirely risk-free, since they can influence driving behavior. Different antihypertensives cause different degrees of performance deficits. Some older antihypertensive drugs such as reserpine, methyldopa, and clonidine are acting in the CNS and can induce sleepiness and hence reduce the ability to drive. Such drugs are only rarely used nowadays. However, modern antihypertensive drugs differ in their influence on driving. McGwin and colleagues [52] investigated the effects of angiotensin-converting enzyme (ACE) inhibitors, beta-blockers, and diuretics on accident rates. After adjusting for age, sex, race, and annual miles driven, the odds of motor vehicle collision were 1.6 (95% CI 1.0, 2.7), 1.4 (95% CI 0.8, 2.3), and 0.9 (95% CI 0.5, 1.7) for ACE inhibitors, beta-blockers, and diuretics, respectively. In a study by Rudisill and colleagues [14], Hydrochlorothiazide, a diuretic, and Metoprolol, a beta-blocker, both slightly increased the risk of a motor vehicle collision. Arora and colleagues [154] compared the effects of losartan, ramipril, and aliskiren (a direct renin inhibitor) on some functions relevant for driving, namely simple response time, multiple-choice response time, flicker fusion frequency, and tracking performance. All three drugs improved the functions examined. This shows that modern antihypertensive drugs may have even positive effects on driving skills.

### 5.2. Cardiac Arrhythmia

Arrhythmias can lead to circulatory disorders in the brain, leading to significant impairment of driving-related functions and reduced alertness. Atrial fibrillation is one of the most prevalent arrhythmias in old age, with a lifetime risk of up to 30% [155], and it often co-exists with arterial hypertension due to common risk factors. Arterial hypertension may also increase the probability of a new atrial fibrillation [156]. Arrhythmias of all types can lead to sudden fainting (syncope) which is a massive risk for driving due to the loss of consciousness. To make matters worse, driving itself can trigger arrhythmias. Driving stress and anger can stimulate the autonomic nervous system, which can increase the risk of an arrhythmia. Air pollution can also increase the risk of arrhythmias [157]. However, the risk is still very low and if adequately treated, e.g., with medication or implanted cardioverter/defibrillator (ICD), further minimized. Curnis and colleagues [158] assessed 286 drivers implanted with ICDs for their risk of MVC compared to the general driving population and found no difference in risk.

### 5.3. Coronary Heart Disease

In old age, the risk of coronary heart disease (CHD) and its possible dramatic consequence of a heart attack increases. According to a retrospective German study, the main cause of sudden natural deaths at the wheel is coronary heart disease [159]. A Canadian autopsy study found that 86% of the drivers above 60 years who died at the wheel had coronary heart disease and 40% of these drivers showed abnormal driving behavior before the accident due to loss of control [160]. To be fit to drive, patients with CHD have to be in a stable state in which the risk of sudden worsening is very low [161]. Some of the patients with CHD experience seizure pain (angina pectoris). Such seizures can be triggered by stress and anger while driving, hence affecting driving performance.

Anticoagulants that are frequently prescribed against arrhythmias and CHD pose a risk of acute bleeding. McGwin and colleagues [52] found a strongly increased accident rate when taking anticoagulants (OR = 2.6). Therefore, drivers treated with anticoagulants should have a careful medical examination which should be repeated regularly.

### 5.4. Heart Failure

Heart failure is mainly a consequence of high blood pressure and coronary artery disease. It affects significantly more old than young people: around 10% of 75 year old, and in particular, men, suffer from heart failure. Heart failure can lead to various symptoms such as cardiac arrhythmia, shortness of breath, dizziness, or mental disorders due to a temporary lack of blood flow to the brain. This often results in a general decrease in physical and mental performance and even a sudden breakdown in performance, which is of course relevant for driving. Older adults with heart failure performed worse than healthy control persons in tests of attention and other cognitive functions and also showed poorer driving performance in the driving simulator [162]. However, driving with heart failure is not necessarily associated with a loss of fitness to drive if the heart failure is not yet pronounced or well treated. For this, the patients must take their medication regularly and adhere to their therapy recommendations.

To summarize, cardiovascular diseases, if well supervised by the attending physician and treated with modern and adequate medication, do not necessarily pose an enhanced risk for driving fitness and accidents in older drivers. Hypertension, which is extremely frequent among older people, is only associated with driving risk if it is extremely high. For professional drivers, the limits are set to lower blood pressure levels. Physicians should measure blood pressure regularly, in particular for older professional drivers, and prescribe modern antihypertensives with only minor negative effects on driving.

## 6. Diabetes Mellitus

Diabetes mellitus is one of the most common diseases of old age. It is estimated that in 2019 one in five people over 65 years suffered from diabetes [163]. Cox and colleagues found an increase in the accident rate of 12–19% in diabetics compared to healthy controls, and, in a further study, an increased number of accidents and self-perception of driving errors in diabetics as well [164,165]. The functional effects caused by diabetes differ depending on the type of diabetes, the duration and severity of the disease, the complications, the main clinical symptoms, the treatment, and the interaction with other diseases. In an international study with about 1000 participants [164], patients with type 1 diabetes had a significantly higher risk of accidents compared to healthy controls, while no such risk enhancement was found for type 2 diabetes.

Road safety in diabetes is primarily impaired due to hypoglycemia which is often induced by the treatment [166,167]. Hypoglycemia can lead to loss of control, behavioral disorders, or impaired consciousness. A study with a very large sample showed that the frequency of accidents in people with hypoglycemia increased up to 5.5% [168]. The risk is highest among commuters and professional drivers, as well as among patients with poor diabetes management [169]. A serious problem with regard to the driving ability of diabetics is a perceptual disorder for hypoglycemia. For example, Stork and co-workers found that patients with type 1 diabetes and hypoglycemia perception disorder often drove cars even with hypoglycemia, while patients with good perception did not [170]. Interestingly, even with good hypoglycemia awareness, patients with type 2 diabetes often made risky driving decisions, especially when taking antidiabetic drugs. However, driving ability can usually be restored by suitable measures such as changes in therapy, training methods to better perceive hypoglycemia, and increased blood sugar self-monitoring, especially before departure [169]. A stable metabolic state with a good perception of hypoglycemia is therefore necessary for driving with diabetes.

Apart from hypoglycemia, diabetics with acute and persistent hyperglycemia can also show cognitive impairments, such as a decrease in attention, concentration, and response speed, which impairs the ability to drive [171].

In diabetics, deficits in working memory were in particular predictive of accidents [172]. Hence, they propose working memory deficits as an indicator to identify diabetics with particularly impaired driving fitness. Since working memory performance deteriorates with age [173], older diabetics may be even more vulnerable than younger patients.

A significant complication of diabetes, which affects about a third of diabetics, is diabetic retinopathy. It is characterized by microaneurysms/hemorrhages, alteration of the blood-retinal barrier, capillary closure, and alterations in the neuronal and glial cells of the retina leading to visual impairment [174]. After 20 years of diabetes, about 90% of the patients suffer from diabetic retinopathy. The consequences are an impairment of visual acuity or color vision as well as a restriction of the field of view [17].

Further, patients with diabetes mellitus are more likely to become frail, meaning to develop sarcopenia, deterioration in muscle and nerve function, declining cardiopulmonary reserve, and reduction of executive function [175]. These comorbid conditions further impair driving fitness.

Different treatments of diabetes are associated with different risks for hypoglycemia. Forms of therapy with a low risk of hypoglycemia include changing the diet and increased physical activity, while antidiabetic agents are associated with different risks depending on the active drug applied. Correctly dosed, antidiabetic drugs such as insulin, biguanide, or sulfonylureas do not adversely affect driving, or can even improve fitness to drive to the level of non-diabetic drivers [176,177]. However, high doses and overdosing of active ingredients may increase the risk of hypoglycemia, which is associated with the above-mentioned impairments of traffic safety.

In summary, well-informed patients who show a stable glucose metabolism are fit to drive. Only if the symptoms are pronounced and recent hypoglycemia needed intervention is driving fitness severely compromised until a stable glucose metabolism is restored. Diabetes is often part or consequence of a wider range of mostly diet- and physical inactivity-related disorders, such as metabolic syndrome (MS). MS is defined as the clustering of abdominal obesity, dyslipidemia, hypertension, and hyperglycemia. It appears to be driving the global epidemics of cardiovascular disease, and each factor itself may be relevant for driving safety.

## 7. Musculoskeletal Disorders

Many older people suffer from diseases of the musculoskeletal system, which are usually accompanied by chronic pain. In the above-mentioned study by Rudinger and colleagues, the most frequently mentioned complaints and diseases were arthrosis, rheumatism, back pain, and slipped discs [7].

Arthrosis, also called osteoarthritis (OA), is very common in the elderly. In OA, the articular cartilage and subsequently the adjacent bones degenerate. The joints in the knees, hips, fingers, and shoulders are mostly affected. In the US, knee OA occurs in 10% of men and 13% of women aged 60 years or older [178]. OA increasingly leads to pain and restricted movement, which impairs activities such as walking and driving. OA in the knee and ankle are particularly relevant for driving since they can slow braking movements. In a driving simulator study, patients with arthrosis in the right hip or knee joint needed longer braking times [179]. Surprisingly, the braking time was also extended for osteoarthritis of the left knee joint. This must be taken into account when evaluating the fitness to drive in knee arthrosis. Special driver assistance systems such as Adaptive Cruise Control (ACC) may be useful to support or improve the braking performance in certain situations. Studies indicate that older drivers use this technology more often than younger ones [21].

In addition to OA, rheumatoid arthritis plays a role in driving ability. Rheumatoid arthritis (RA) is an inflammatory autoimmune disease of the joints, which is accompanied by overheating, swelling, and redness, as well as restricted movement and pain. These symptoms can affect driving activities because of difficulties with braking due to swollen and/or tender foot joints [180], or difficulties with looking to the left or right due to neck pain [181]. The latter could be one reason why many older drivers do not perform shoulder checks, even in situations where these would be necessary [182]. In several studies, RA patients reported illness-related driving difficulties or even a complete inability to drive [181,183]. A recent review collected evidence on whether and how RA has an impact on fitness to drive and driving safety [184]. The authors found some studies that show an increased number of motor vehicle crashes (MVCs) in RA patients, while Orriols and colleagues [185] reported that less than 50% of the RA patients who were involved in an accident were responsible for the crash.

Even after surgery and joint prostheses, restrictions in fitness to drive may persist for some time. For example, Jordan and colleagues [186] found that after a knee joint replacement, the braking time was prolonged and that the initial values before the operation were only reached again after six weeks. Patients should therefore not restart driving until six weeks after knee joint surgery. Interestingly, in a study of Talusan and colleagues [187], all 37 patients with foot and ankle pathology receiving local anesthetic and steroids through image-guided injection reported a more than 90% pain relief post-injection. Despite symptom improvement, there was no significant change in brake reaction time irrespective of the side of the leg affected. We can only speculate whether fear of recurrent pain and setback of symptoms or procedural habituation might have played a role.

Some of the movement restrictions in musculoskeletal disorders can be probably mitigated by using modern driver assistance systems, such as the blind spot warner, which can compensate for limited agility in the shoulder and neck area and make changing lanes safer. This technology is used by many older drivers [188] and usually rated as useful [189]. Parking assistants can be helpful, as they facilitate parking via sensors and reverse cameras or maneuver the car semi-autonomously into a parking space [21].

In addition to the restrictions on movement, the pain that almost always occurs with diseases of the musculoskeletal system can have unfavorable effects on driving behavior. Pain can have a distracting effect on the driver and is associated with impaired psychomotor abilities such as lane- keeping or cognitive performance such as sustained attention [190,191]. Many drivers take analgesics to reduce pain. In addition to their pain-relieving effects, these often have side effects, which in turn can impair driving [13]. Opioid analgesics, which can significantly reduce the ability to drive, are particularly critical. Their side effects include impairment of muscle coordination, as well as sensory and cognitive performance, e.g., impaired concentration, as well as drowsiness and sedation. Studies show that the crash risk increases significantly after taking opiates as a pain reliever [129,192]. A recent review puts the odds ratio (OR) for the risk of being involved in an accident under the influence of prescription opioids up to 8.2 and the risk of causing an accident up to 2.78 [6]. There seems to be an association between opioid use and several self-reported measures of driving behavior and ability including self-regulation and reduction of driving, as well as lower self-rated driving ability. However, in the American LongROAD study, no association between opioid use and motor vehicle crashes could be found [193].

High doses of non-opioids also interfere with driving safety, as they may cause undesirable side effects such as nausea, vomiting, tiredness, and dizziness. Non-opioids can also impair psychomotor skills relevant to driving. For example, indomethacin and phenylbutazone (but not acetylsalicylic acid) affected eye-hand coordination and divided attention up to 150 min after their administration [194]. Some authors found a significantly increased accident rate after taking non-steroidal anti-inflammatory drugs (e.g., [52]). In particular, when analgesics are started or adjusted to higher dosages, doctors have to bear in mind and advise the patient that his/her driving fitness is likely to be compromised [14].

In summary, diseases of the musculoskeletal system, in particular knee arthrosis, may impair driving behavior. Even after surgery and joint prostheses, restrictions in fitness to drive may persist for some time. Modern advanced driver assistance systems (ADAS) may help when movements are restricted or painful. Analgesics often prescribed against pain are likely to impair driving safety, in particular opioids. Physicians should be aware of the high risks of opioids for drivers.

## 8. Frailty

Frailty is a syndrome following a decline in function and resilience across numerous physiological systems [195]. It is defined as “a clinical state in which there is an increase in an individual’s vulnerability for developing increased dependency and/or mortality when exposed to a stressor” [196]. One commonly used frailty measure is the frailty phenotype created and validated by Fried and colleagues, which provides a standardized measure based on the assessment of five criteria: unintentional weight loss, weakness, exhaustion, slowness, and low physical activity [197]. An older person is classified as frail when three or more of these five components are present; pre-frailty is classified when one or two components exist. Frailty among older adults is associated with an increased risk of adverse health outcomes, such as falls, incident disability, hospitalization, and crash-related injury or death [197]. In the LongROAD study, the association of frailty with reduced driving space and involvement in motor vehicle crashes was studied. Pre-frail participants had 30% higher odds of reporting involvement in a crash in the prior year than non-frail participants after adjusting for several confounding factors [198]. In a recent Japanese study [199], 349 older drivers were classified in a (pre-)frail and a robust group. Pre-frailty/frailty was associated with more near-miss traffic incidents compared to robustness. Pre-frail/frail participants showed higher rates of traffic crash involvement in the past year than robust participants. These studies clearly show an enhanced accident risk for pre-frail and frail drivers.

Crowe and colleagues [195] studied the association between frailty status and low mileage driving and driving cessation respectively in almost 3000 subjects. They outlined that for every unit increase in frailty, the estimated risk of driving fewer than 1865 miles/year increased by 138%. Relative to older drivers who were not frail, the adjusted hazard ratios of driving cessation were 4.15 (95% CI: 1.89–9.10) for those classified as prefrail and 6.08 (95%CI: 1.36–27.26) for those classified as frail. The authors concluded that frailty is positively associated with low-mileage driving status and driving cessation in a dose-response fashion. A better functioning of the lower extremities (measured by the Short Physical Performance Battery, SPPB) is related to driving longer distances and to lower crash rates in 65–79-year-old drivers [200]. Given that continued mobility is essential to maintaining one’s ability to remain integrated into society and complete the tasks required for daily living, low-mileage driver status and driving cessation will present a major threat to physical, social, and mental health for many of these older adults, especially those who lack access to alternative transportation.

Old age and in particular frailty cause an enhanced vulnerability to injury in the event of a crash [199,201], such as fatal chest and head injuries [202].

## 9. Polypharmacy and Driving Fitness

A widely present problem among elderly drivers is polypharmacy. Due to multi-morbidity, commonly defined as the co-existence of two or more chronic health conditions, the usage of multiple medicines is common in the older population. Polypharmacy is often referred to as being on five or more medicines and is associated with adverse outcomes including mortality, falls, adverse drug reactions, increased length of stay in hospital, etc. [203]. The risks of polypharmacy include an increased number of potentially driver impairing medications (PDI). Both multi-morbidity and polypharmacy have a major impact on being able to safely steer a car in heavy traffic. Each factor itself, chronic disease as well as the medication administered, may impact driving safety, adding up with each further disorder or medical substance. LeRoy & Morse [15] published a very thorough analysis of multiple medications and vehicle crashes in older people and found a significant association between PDI and crashes. Many combinations included interacting substances that potentiate adverse effects on driving, such as narcotic analgesics with muscle relaxants, antidepressants, or antianxiety agents. Disease-medication relationships are difficult to estimate, as to whether the disease itself or the interplay with the medication or its side effects are more relevant to interfere with driving fitness. A patient suffering from a disease that is well-controlled by medication is more likely to drive safely than a patient with the same but untreated disease. Interaction between medications often remains undetected or underestimated, although internet-based databases and international recommendations for medication e.g., the DRUID categorization [192] and PRISCUS-list [204] can assist in choosing the most appropriate medication. They provide information about appropriate and non-appropriate substances for older people and in the context of driving. However, recent publications suggest that polypharmacy may also be a risk factor for under-prescribing, such that patients do not receive necessary medications if they are on “too many drugs” and this can also pose risks to patients’ (driving) safety and well-being. For example, Kuijpers and colleagues [205] showed that a significantly higher proportion of patients receiving polypharmacy were undertreated (42.9%) compared with those receiving fewer medicines (13.5%). Hence, polypharmacy is “potentially problematic rather than always inappropriate” [206]. Medication adherence is another important clinical issue in ensuring safe and effective medicine use as it has been estimated that 50% of patients do not take their medications correctly. Polypharmacy is in particular associated with medication non-adherence in older people.

## 10. General Recommendations

The age-related diseases and disorders mentioned in this review and the corresponding medical treatment may substantially affect driving behavior and traffic safety. Moreover, physicians increasingly have to care for old and very old patients suffering from numerous medical conditions under polypharmacy. The degree of safety impairment depends on the disease and its stage, and the choice of the proper medication. International recommendations and internet-based databases may assist in making the best choice of medication suitable for each patient. However, physicians sometimes tend to overestimate the fitness to drive of their patients [207], which may be due to fear of harming the doctor-patient relationship, ambiguous fitness to drive criteria, or uncertainty in legal and ethical obligations [37].

What can be advised to physicians? First, they should see patients with the mentioned age-related diseases regularly. They should properly test the patients for sensory, motor, and cognitive impairments that are relevant for driving. Simple tests of visual acuity, neck rotation, or reaction time are better than nothing but clearly not sufficient. For testing, physicians should seek cooperation with specialized colleagues such as ophthalmologists and psychiatrists. In case of clear risks for driving safety, general practitioners should refer patients to specialists, which also helps to keep a good doctor–patient relationship. Physicians should be trained on how to counsel their patients and their relatives concerning disease-related impairments of driving safety due to high age and age-related diseases, and to relevant side effects of medication. Furthermore, the patients should be advised to make use of specific behavioral strategies, car technologies, and interventions to maintain driving fitness as long as possible. Strategies comprise the avoidance of difficult driving situations, such as night driving. During driving, attention-directed strategies such as the preparation for critical situations are useful. Technologies comprise the use of in-vehicle information systems (IVIS) or advanced driver assistance systems (ADAS). In particular, route guidance systems are most helpful for older drivers since they reduce visual search. Also important are sensors that signal distance from obstacles and parking assistants that support backing into a parking space [3]. Interventions, namely cognitive training, may help to restore aspects of attention, working memory as well as spatial perception, orientation, and reaction time [3]. Likewise, physical exercise may help to improve general physical fitness, strengthen muscles, normalize the muscle tone, and improve head and neck flexibility as well as motor coordination. Further, on-road driving lessons with a trained driving instructor may depict shortcomings in driving behavior and help to overcome them or show compensation strategies [3]. As a consequence of a thorough assessment, driving fitness restrictions or recommendations may apply to the patient as e.g., driving only during the daytime. Finally, if driving is no longer possible (e.g., in dementia), strategies to improve the accessibility of alternative sources of transportation should be discussed with the patients and their relatives.

To face the challenge of a significantly rising number of older drivers and those with age-related diseases, clinical, technical, and political stakeholders should cooperate. From a technical perspective, IVIS and ADAS should be optimized for, and tested with, older and disabled drivers, thereby increasing their driving safety and comfort [21]. IVIS should give announcements sparsely and just in time for proper preparation. The development of more intelligent systems for older and disabled drivers requires further research.

From a political perspective, a statutory framework would help physicians as well as patients and their families to deal with medical conditions in the context of aging and driving fitness, e.g., through compulsory biennial neurocognitive and physical testing beyond the age of 70 years or an obligation to seek medical counseling for patients with medical conditions very likely to affect driving fitness, such as dementia.

To conclude, with an aging population, traffic safety is a more and more prevalent concern on roads of industrialized countries. We tried to give an overview of the most relevant research on age-related disorders as well as related medication affecting driving fitness in the elderly, along with recommendations for physicians.
